# Association between the American Heart Association’s new “Life’s Essential 8” metrics and kidney stone

**DOI:** 10.1007/s00345-024-04867-9

**Published:** 2024-03-27

**Authors:** Xingmo Dong, Lihua Liao, Yani Wang, Xueqin Lin, Weihua Chen, Huaijing Luo, Yi Yi, Dewen Zhong, Haifeng Wang, Zecong Ma, Yongfei Liu, Ying Liao

**Affiliations:** 1 Longyan First Affiliated Hospital of Fujian Medical University, Longyan, 364000 China; 2 The Third Clinical Medical College, Fujian Medical University, Fuzhou, 350000 China; 3 Beijing Friendship Hospital, Capital Medical University, Beijing, 100053 China

**Keywords:** Cardiovascular health, Life’s Essential 8 (LE8), National Health and Nutrition Examination Survey (NHANES), Kidney stone

## Abstract

**Purpose:**

The incidence of kidney stone disease has increased worldwide, resulting in high medical costs and social burden. Kidney stone disease shares some common features with the risk factors of cardiovascular diseases (CVDs). We investigated the association between cardiovascular health (CVH) based on the Life’s Essential 8 (LE8) score developed by the American Heart Association and the incidence of kidney stone disease.

**Methods:**

We analyzed the data of 29,469 US adults aged 20 years or above from the National Health and Nutrition Examination Survey, 2007–2018. According to the LE8 score, CVH was divided into three categories: poor, intermediate, and ideal. Logistic regression was used to determine the association between CVH and the incidence of kidney stone disease by estimating odds ratios (ORs) and 95% confidence intervals (CIs).

**Results:**

The average age of the participants was 48.6 years, and 50% of the participants were women. The numbers of participants with poor, intermediate, and ideal CVH were 4149, 19,782, and 5538, respectively. After adjusting for related confounding factors, ideal CVH was associated with a reduction in the odds of kidney stone occurrence as compared to poor CVH (adjusted OR [aOR]: 0.45, 95% CI: 0.35–0.57, *p* < 0.001). Moreover, if the ideal CVH metrics was ≥ 6, the odds of kidney stone occurrence decreased by up to 61% (aOR: 0.39, 95% CI: 0.30–0.51).

**Conclusions:**

In the present study, ideal CVH, a factor indicative of a healthy lifestyle, was associated with lower odds of kidney stone occurrence.

**Supplementary Information:**

The online version contains supplementary material available at 10.1007/s00345-024-04867-9.

## Introduction

Kidney stone disease is one of the most prevalent diseases worldwide, and its prevalence in all age, gender, and ethnic groups has been increasing over the past few decades [1]. The high prevalence and recurrence rates of kidney stone disease place a heavy burden on the global economy. According to recent statistics, the United States government alone spends more than $2 billion annually on the healthcare of patients with kidney stones [2].

Previous studies have shown that the risk of kidney stone occurrence increases with the occurrence of diabetes, obesity, and hypertension and poor diet [3, 4]. Patients with kidney stones also have a significantly higher risk of cardiovascular events, including coronary heart disease (CHD), acute myocardial infarction, and chronic kidney disease [5, 6].

In 2010, the American Heart Association (AHA) quantified cardiovascular health (CVH) into seven desirable metrics [7]. In 2022, the AHA developed a new scoring method for CVH with an updated version of “Life’s Essential 8” (LE8) metrics, which included diet, physical activity, nicotine exposure, sleep health, body mass index, blood lipids, blood glucose, and blood pressure [8]. Over the past 12 years, the application of the ideal CVH status not only improved the poor prognosis of patients with cardiovascular diseases (CVDs) but also reduced the risk of type 2 diabetes, dementia, and sarcopenia in these patients [9, 10]. However, the relationship between the ideal CVH status and the risk of kidney stone occurrence has not yet been clarified.

Therefore, the present study aimed to investigate the association of the ideal CVH status as determined by the LE8 score and the prevalence of kidney stone disease in a national sample of US men and women.

## Materials and methods

### Participant details

The National Health and Nutrition Examination Survey (NHANES) is a nationally representative survey designed and administered by the National Center for Health Statistics (NCHS) to assess the health and nutritional status of the US household population. The project surveys a nationally representative sample of approximately 5000 people at approximately every 2 years, and it covers a variety of health and nutrition measures. Written informed consent was obtained from all participants, and the study was approved by the NCHS Ethics Review Committee (protocol numbers: NHANES Continuation of Protocol #2005-06, NHANES Protocol #2011-17, and NHANES Protocol #2018-01).

**Fig. 1:**
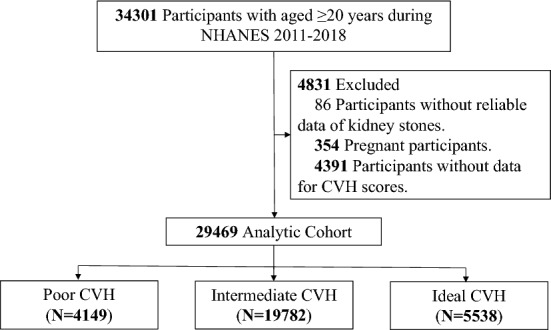
Flow chart. *NHANES* The National Health and Nutrition Examination Survey

As shown in the Fig. 1, this study included participants ≥ 20 years of age who were enrolled in NHANES 2007–2018 (*n* = 34,301). Of these participants, 4832 were excluded based on the following criteria: (1) pregnancy, (2) insufficient data to diagnose kidney stones, and (3) insufficient data to calculate the CVH scores. Thus, 29,469 participants were finally enrolled in the present study.

### Diagnosis of kidney stones

Results of the questionnaire determines whether the participant has a history of nephrolithiasis. If the answer to the question “Have you/Has sample person (SP) ever has a kidney stone?” If a participant answered yes to ever had a kidney stone, he was considered to have kidney stone.

### CVH metrics

CVH indicators include four health behaviors (diet, physical activity, nicotine exposure, and sleep) and four health factors (body mass index, blood lipids, blood glucose, and blood pressure). The Healthy Eating Index 2015 (HEI-2015) scores were used as a proxy for healthy eating scores and were calculated based on a 24-h dietary recall on the first day. HEI-2015 is a dietary index based on 13 components, which are combined to yield a total score of up to 100 points, with higher scores indicating a healthier diet. Among the 13 components, nine components assess dietary adequacy (total fruit, whole fruit, total vegetables, greens and beans, whole grains, dairy products, total protein foods, seafood and vegetable proteins and fatty acids) and the remaining three components examine dietary moderation (refined grains, sodium, added sugars and saturated fats). For determining sleep health, objective sleep data from wearable gadgets were used to determine the average number of sleep hours, and the score was calculated accordingly. Nicotine exposure was scored by self-reported use of cigarettes or electronic nicotine delivery systems (ENDS); the adverse health effects of secondhand smoke were also included in the score.

Glycated hemoglobin (HbA1c) or fasting blood glucose level was used as a blood glucose score. Prior history of diabetes was confirmed for all participants before scoring. Non-high-density lipoprotein cholesterol, instead of total cholesterol, was used to score blood lipids. BMI was calculated by dividing body weight in kilograms by the square of height in meters. The mean blood pressure was estimated from up to 3 readings obtained under standard conditions during a single physical examination. The details of scoring for each metric are provided in Supplemental Table S1.

For each metric, the participants received a score of 0–100, and the scores were assigned as follows: < 50, poor health; 50–79, moderate health; and ≥ 80, ideal health. CVH was calculated as the unweighted average score of all eight metrics, with a total rating of 0–100. Participants with an average score of 0–49, 50–79, or 80–100 were categorized to have poor, intermediate, or ideal CVH, respectively.

### Variables of interest

Age, sex, race, education level, alcohol use, marital status, income, CHD, and cancer history were self-reported. We performed analyses using CVH that was determined based on the definition and scoring approach for quantifying CVH in accordance with the AHA’s LE8 score [8]. The participants were assigned to three CVH categories: (1) poor CVH with scores of 0–49; (2) intermediate CVH with scores of 50–79; and (3) ideal CVH with scores of 80–100.

### Statistical analysis

NHANES-recommended weights were used to account for the planned oversampling of specific groups. Continuous variables were expressed as mean ± standard deviation. Categorical variables were expressed as counts (percentages). Baseline characteristics between three CVH groups were compared using a *t*-test for continuous variables and chi-squared test for categorical variables. Multiple logistic regression was used to examine the association between CVH metrics and kidney stones after adjustments for potential confounders such as age, sex, race, education levels, alcohol use, marital status, income, CHD, and cancer. The odds ratio (OR) and 95% confidence interval (CI) were estimated. We determined the relationship between CVH and kidney stones in a subgroup analysis based on sex and separately assessed the association between individual components of the CVH metrics and kidney stones. We also used multiple logistic regression analysis to assess the effect of several ideal cardiovascular health metrics (ICVHMs) on the incidence of kidney stones. A two-sided *p* value of < 0.05 indicated statistical significance for all analyses. All data analyses were performed using SAS version 9.4 (SAS Institute) and survey package in R software (version 4.0.4; R Foundation for Statistical Computing, Vienna, Austria).

## Results

### Patient characteristics

The mean age of the 29,469 participants was 48.45 ± 0.26 years, and approximately 50% of the participants were females. The proportion of participants with poor, intermediate, and ideal CVH was 14.1%, 67.1%, and 18.8%, respectively. In the present sample, the frequency of kidney stone occurrence in participants with poor, moderate, and ideal CVH was 15.05%, 11.26%, and 5.23%, respectively. The baseline characteristics of the study population are shown in Table 1.

**Table 1:** Baseline characteristics of the study population

Characteristic	Overall *N* = 29,469	Poor *N* = 4149	Intermediate *N* = 19,782	Ideal *N* = 5538	*p* Value
Age, years	48.45 (0.26)	55.00 (0.39)	49.78 (0.27)	41.35 (0.43)	< 0.0001
BMI, kg/m^2^	29.32 (0.09)	35.32 (0.24)	29.92 (0.10)	24.58 (0.07)	< 0.0001
TC, mg/dL	192.49 (0.53)	207.46 (1.24)	194.33 (0.67)	179.64 (0.76)	< 0.0001
HDL, mg/dL	52.93 (0.22)	45.37 (0.36)	51.48 (0.21)	60.91 (0.41)	< 0.0001
HbA1c, %	5.73 (0.01)	6.67 (0.04)	5.78 (0.01)	5.26 (0.01)	< 0.0001
SBP, mmHg	122.42 (0.22)	134.50 (0.61)	123.73 (0.24)	112.60 (0.25)	< 0.0001
DBP, mmHg	70.77 (0.20)	73.98 (0.40)	71.29 (0.21)	67.65 (0.28)	< 0.0001
Female, *n* (%)	14,907 (50.59)	2199 (53.08)	9496 (48.24)	3212 (58.27)	< 0.0001
Ethnicity, *n* (%)					< 0.0001
Black	6110 (20.73)	1198 (16.10)	4182 (11.21)	730 (6.62)	
Mexican American	4527 (15.36)	607 (8.03)	3170 (9.01)	750 (7.61)	
White	12,442 (42.22)	1678 (64.36)	8322 (66.64)	2442 (69.80)	
Other	6390 (21.68)	666 (11.51)	4108 (13.14)	1616 (16.00)	
Alcohol use, *n* (%)					< 0.0001
No	6142 (20.84)	869 (17.05)	4079 (16.65)	1194 (16.58)	
Now	18,789 (63.76)	2205 (57.95)	12,639 (70.80)	3945 (77.38)	
Former	4538 (15.40)	1075 (25.00)	3064 (12.55)	399 (6.04)	
Education level, *n* (%)					< 0.0001
< 12	7184 (24.40)	1517 (27.12)	4940 (16.22)	727 (7.72)	
12	6742 (22.90)	1080 (29.41)	4812 (25.55)	850 (13.59)	
> 12	15,521 (52.71)	1551 (43.47)	10,010 (58.22)	3960 (78.69)	
Kidney stone, *n* (%)	3034 (10.30)	627 (15.05)	2112 (11.26)	295 (5.63)	< 0.0001
Marital status, *n* (%)					< 0.0001
Married	17,639 (59.87)	2291 (58.97)	11,960 (63.28)	3388 (63.04)	
Divorce/separated/widowed	6690 (22.71)	1354 (27.74)	4661 (20.35)	675 (10.55)	
Never married	5131 (17.42)	502 (13.29)	3155 (16.36)	1474 (26.41)	
Income, *n* (%)					< 0.0001
< $20,000	8205 (27.84)	1613 (31.78)	5452 (20.40)	1140 (16.21)	
≥ $20,000	21,264 (72.16)	2536 (68.22)	14,330 (79.60)	4398 (83.79)	
Smoke, *n* (%)	13,167 (44.68)	2954 (74.57)	9224 (48.20)	989 (20.22)	< 0.0001
Cancer, *n* (%)	2956 (10.04)	453 (11.60)	2135 (11.78)	368 (7.64)	< 0.0001
CVD, *n* (%)	3690 (12.52)	1048 (22.44)	2435 (10.15)	207 (3.29)	< 0.0001

*BMI* body mass index; *TC* total cholesterol; *HDL* high-density lipoprotein; *HbA1c* hemoglobin A1c; *SBP* systolic blood pressure; *DBP* diastolic blood pressure; *CVD* cerebrovascular disease

### Association between CVH score, number of ICVHMs, and kidney stone

Ideal CVH was associated with a reduction in the odds of kidney stone occurrence as compared to poor CVH (OR: 0.34, CI: 0.27–0.42, *p* < 0.001; Table 2). After adjusting for age, sex, race, education levels, heavy alcohol consumption, marital status, income, CHD, and cancer, ideal CVH was still associated with a reduction in the odds of kidney stone occurrence as compared to poor CVH (adjusted OR [aOR]: 0.45, 95% CI: 0.35–0.57, *p* < 0.001). Logistic regression analysis of the number of ICVHMs and the odds of kidney stone occurrence revealed that the higher the number of ICVHMs, the lower was the odds of kidney stone occurrence. Compared to participants with non-ideal CVH scores, those with ideal CVH scores showed a 45% decrease in the odds of kidney stone occurrence (aOR: 0.55, 95% CI: 0.42–0.72). If the number of ICVHMs was ≥ 6, the odds of kidney stone occurrence decreased by up to 61% (aOR: 0.39, 95% CI: 0.30–0.51; Table 2). Subgroup analysis showed that in most subgroups, the ideal CVH metrics group was associated with a lower risk of kidney stone occurrence (Table 3). For the elder (aOR: 0.44, 95% CI: 0.33,0.59, *p* < 0.0001) and female (aOR: 0.41, 95% CI: 0.28–0.60, *p* < 0.0001) patient, a reduction in the odds of kidney stone occurrence.

**Table 2:** The association between CVH metrics and number of ICVHMs and kidney stone

Categories	Model 1		Model 2		Model 3	
	HR (95% CI)	*p* Value	HR (95% CI)	*p*-Value	HR (95% CI)	*p* Value
*CHV*						
Poor	Ref	Ref	Ref	Ref	Ref	Ref
Intermediate	0.72 (0.60–0.86)	< 0.0010	0.74 (0.62–0.90)	0.0030	0.79 (0.64–0.96)	0.0200
Ideal	0.34 (0.27–0.42)	< 0.0001	0.41 (0.33–0.52)	< 0.0001	0.45 (0.35–0.57)	< 0.0001
*Number of ICVHMs*						
≤ 1	Ref	Ref	Ref	Ref	Ref	Ref
2	0.97 (0.77–1.23)	0.8200	0.98 (0.77–1.24)	0.8400	0.98 (0.77–1.25)	0.8700
3	0.73 (0.60–0.90)	0.0040	0.75 (0.60–0.93)	0.0100	0.76 (0.60–0.96)	0.0200
4	0.64 (0.50–0.83)	< 0.0010	0.70 (0.54–0.91)	0.0100	0.74 (0.56–0.97)	0.0300
5	0.44 (0.34–0.57)	< 0.0001	0.52 (0.40–0.67)	< 0.0001	0.55 (0.42–0.72)	< 0.0001
≥ 6	0.28 (0.22–0.37)	< 0.0001	0.37 (0.28–0.48)	< 0.0001	0.39 (0.30–0.51)	< 0.0001

Model 1: unadjusted

Model 2: adjusted for age, gender, race

Model 3: adjusted for age, gender, race, education levels, alcohol use, marital, income, coronary heart disease, cancer

**Table 3:** Adjusted ORs (95% CI) of kidney stone by subgroup of CVH metrics

Subgroup	Adjusted OR^a^ (95%CI)	*p* Value
*Sex*		
Male		
Poor	Ref	Ref
Intermediate	0.74 (0.57, 0.97)	0.03
Ideal	0.46 (0.33, 0.65)	< 0.0001
Female		
Poor	Ref	Ref
Intermediate	0.82 (0.61, 1.10)	0.19
Ideal	0.41 (0.28, 0.60)	< 0.0001
*Age*		
< 60		
Poor	Ref	Ref
Intermediate	0.96 (0.71, 1.29)	0.77
Ideal	0.56 (0.38, 0.83)	0.004
≥ 60		
Poor	Ref	Ref
Intermediate	0.75 (0.59, 0.96)	0.02
Ideal	0.44 (0.33, 0.59)	< 0.0001
*Ethnicity*		
Mexican American		
Poor	Ref	Ref
Intermediate	0.83 (0.54, 1.28)	0.39
Ideal	0.60 (0.29, 1.26)	0.18
Black		
Poor	Ref	Ref
Intermediate	0.72 (0.48, 1.08)	0.11
Ideal	0.50 (0.25, 1.00)	0.05
White		
Poor	Ref	Ref
Intermediate	0.79 (0.61, 1.02)	0.07
Ideal	0.47 (0.35, 0.64)	< 0.0001
Other		
Poor	Ref	Ref
Intermediate	0.75 (0.49, 1.16)	0.2
Ideal	0.26 (0.16, 0.43)	< 0.0001
*Education level*		
< 12		
Poor	Ref	Ref
Intermediate	0.73 (0.54, 0.99)	0.04
Ideal	0.42 (0.24, 0.72)	0.002
12		
Poor	Ref	Ref
Intermediate	1.02 (0.70, 1.51)	0.9
Ideal	0.66 (0.40, 1.10)	0.11
> 12		
Poor	Ref	Ref
Intermediate	0.71 (0.53, 0.96)	0.03
Ideal	0.40 (0.29, 0.55)	< 0.0001

^a^Analyses were adjusted for age, gender, race, education levels, alcohol use, marital, income, coronary heart disease, cancer

### Association between individual CVH components and kidney stone occurrence

Table 4 shows the adjusted ORs (95% CI) for kidney stone occurrence according to individual CVH components for poor, intermediate, and ideal CVH. Participants with intermediate or poor CVH had a higher odds of kidney stone occurrence than those with ideal CVH in the following CVH metric subgroups: diet, physical activity, sleep, BMI, and blood glucose. In general, individual health behaviors were associated with kidney stone occurrence.

**Table 4:** Adjusted ORs (95% CI) of kidney stone by individual component of CVH metrics

Subgroup	Adjusted OR^a^ (95%CI)	*p*-Value
Diet		
Poor	Ref	Ref
Intermediate	0.77 (0.66, 0.89)	< 0.0010
Ideal	0.64 (0.55, 0.74)	< 0.0001
Physical activity		
Poor	Ref	Ref
Intermediate	1.00 (0.70, 1.44)	1.0000
Ideal	0.79 (0.68, 0.91)	0.0020
Smoke		
Poor	Ref	Ref
Intermediate	0.98 (0.80, 1.19)	0.8100
Ideal	0.93 (0.79, 1.09)	0.3800
Sleep		
Poor	Ref	Ref
Intermediate	0.95 (0.76, 1.19)	0.6500
Ideal	0.73 (0.60, 0.89)	0.0020
BMI		
Poor	Ref	Ref
Intermediate	0.73 (0.62, 0.85)	< 0.0010
Ideal	0.54 (0.47, 0.63)	< 0.0001
Blood lipids		
Poor	Ref	Ref
Intermediate	1.05 (0.87, 1.27)	0.6100
Ideal	0.97 (0.84, 1.11)	0.6300
BP		
Poor	Ref	Ref
Intermediate	0.91 (0.76, 1.08)	0.2800
Ideal	0.91 (0.76, 1.09)	0.3000
Blood glucose		
Poor	Ref	Ref
Intermediate	0.70 (0.57, 0.85)	< 0.0010
Ideal	0.50 (0.42, 0.58)	< 0.0001

^a^Analyses were adjusted for age, gender, race, education levels, alcohol use, marital, income, coronary heart disease, cancer

*BMI* body mass index; *BP* blood pressure

## Discussion

To the best of our knowledge, the present study is the first to confirm that the American Heart Association’s New “Life’s Essential 8” Metrics is significantly associated with the incidence of kidney stones. Our results indicated that the risk of kidney stones was reduced by 61% if the number of ideal CVH metrics was ≥ 6. Following adjustment for potential confounders, higher and ideal CVH remained positively associated with a reduced risk of kidney stone occurrence.

Some previous studies have shown that CVH metrics were associated not only with CVDs but also with non-CVDs, including kidney stones [9, 11, 12]. This observation agreed with the results of Domingos et al. who found that kidney stones was significantly associated with an increased risk of developing CVDs such as CHD and hypertension [13]. Moreover, studies using the NHANES data also observed that obesity and sleep deprivation were associated with higher odds of kidney stone occurrence [14, 15]. However, a comprehensive evaluation of the relationship between cardiovascular risk factors and kidney stones is lacking. All the ideal CVH metrics are optimum to explore this relationship. We found that the increase in CVH score is associated with a low possibility of kidney stone occurrence; this finding suggests a close relationship between CVH and non-CVDs. in future, we can attempt to improve the diet pattern and control the levels of blood glucose and blood lipids to achieve or maintain high CVH and reduce the incidence of CVDs and kidney stones, thereby reducing medical costs.

Our results suggest that CVH affects the incidence of kidney stone regardless of gender, age and ethnicity. We also attempted to determine the effect of individual CVH components on kidney stone occurrence. The results showed that a healthy diet is associated with a reduced risk of kidney stone occurrence. This finding is not surprising, as previous studies have shown that a higher intake of proteins, fats, and sodium is associated with a higher risk of kidney stone occurrence, in contrast to the beneficial effects of a healthy diet rich in fruits and vegetables [16–18]. The association of a lower BMI with kidney stone disease is the second important feature. Eiichi Yoshimura et al. reported that obesity is an independent risk factor for kidney stone disease [19, 20]. Several possible mechanisms could explain the beneficial effect of a lower BMI on kidney stone occurrence. First, the excretion rate of calcium, oxalic acid, and uric acid is higher in the urine of overweight people, and the urine is in a supersaturation state; this can easily lead to the accumulation of stones in the kidney [21, 22]. In addition, overweight people often like to eat foods rich in proteins, fats, and sodium, which affect the metabolism and pH of the kidney and subsequently contribute to the occurrence of stones [23, 24]. Increased HbA1c levels were significantly associated with kidney stone occurrence [25]. This might be because of the same metabolic abnormalities found in kidney stones and diabetes; moreover, poor blood glucose control and insulin resistance can lead to increased excretion of urinary calcium and uric acid, which are closely associated with the increased incidence of kidney stones [26, 27]. Metabolic syndrome is thought to increase the rate of kidney stone formation [27, 28]. However, it is interesting to note that blood lipids and blood pressure, which are associated with the occurrence of metabolic syndrome, did not significantly affect the development of kidney stones. The underlying mechanisms remain unclear and need to be confirmed by further studies.

The results of the present study have several clinical implications. First, clinical and public health professionals need to focus more on CVH metrics to reduce the occurrence of kidney stones and other diseases through lifestyle behavior interventions. Second, public health workers should formulate policies to improve CVH in community populations. Our study on the relationship between kidney stones and CVH metrics further adds to the vital role of CVH in controlling the occurrence of kidney stones.

This study has several limitations. First, this study was retrospective in nature, and although most of the potential confounding factors were adjusted in the multivariate analysis, it is impossible to control for all confounding factors. Second, because of the limitations of the cross-sectional NHANES data, we were unable to prove the causal relationship but could only confirm the association between CVH and kidney stones. Third, smoking, physical activity, disease, and diet were self-reported, and there was a risk of over-reporting or under-reporting of these factors. Fourth, the conclusions were derived from a national survey in the United States, and hence, they may not apply to other ethnic populations.

## Conclusion

In this retrospective cohort study, we found that adherence to a healthy lifestyle identified by the CVH metrics may reduce the incidence of kidney stones. Our analysis extends the findings of previous studies by showing that interventions to prevent CVDs are also promising to prevent the occurrence of kidney stones.

## Supplementary Information

Below is the link to the electronic supplementary material.

Supplementary file1 (DOCX 15 KB)

## Data Availability

The datasets supporting the conclusions of this article are available in the National Health and Nutrition Examination Survey repository [https://www.cdc.gov/nchs/nhanes/].
