# Supplementary material for: Association between the American Heart Association’s new “Life’s Essential 8” metrics and kidney stone
Source: World J Urol. 2024 Mar 27;42(1):199. doi: 10.1007/s00345-024-04867-9 (PMC10973081; doi:10.1007/s00345-024-04867-9)
Supplement: Supplementary file 1 — Supplementary file1 (DOCX 15 KB) [file 345_2024_4867_MOESM1_ESM.docx]

**Supplemental Table S1**. Definition and scoring approach for quantifying cardiovascular health, as per the American Heart Association’s Life’s Essential 8 score (35766027), and as applied in the National Health and Nutrition Examination Surveys, 2007-2018.

| **Domain** | **CVH Metric** | **Method of Measurement** | **Quantification of CVH Metric(≥20 Years)** |
| --- | --- | --- | --- |
| **Health Behaviors** | **Diet** | **Measurement**: Self-reported daily intake of a DASH-style eating pattern  **Example tools for measurement**: DASH diet score (populations) | Quantiles of DASH-style diet adherence  **Scoring (Population)**:  Points Quantile  100 ≥95^th^ %ile (top/ideal diet)  80 75^th^ – 94^th^ %ile  50 50^th^ – 74^th^ %ile  25 25^th^ – 49^th^ %ile  0 1^st^ – 24^th^ %ile (bottom/least ideal quartile) |
|  | **Physical activity** | **Measurement**: Self-reported minutes of moderate or vigorous physical activity per week  **Example tools for measurement**:  NHANES PAQ-K questionnaire | **Metric**: Minutes of moderate (or greater) intensity activity per week  **Scoring**:  Points Minutes  100 ≥150  90 120 – 149  80 90 – 119  60 60 – 89  40 30 – 59  20 1 – 29  0 0 |
|  | **Nicotine exposure** | **Measurement**: Self-reported use of cigarettes or inhaled nicotinedelivery system  **Example tools for measurement**:  NHANES SMQ | **Metric**: Combustible tobacco use and/or inhaled NDS use; or secondhand smoke exposure  **Scoring**:  Points Status  100 Never smoker  75 Former smoker, quit ≥5 yrs  50 Former smoker, quit 1 - <5 yrs  25 Former smoker, quit <1 year, or currently using inhaled NDS  0 Current smoker  Subtract 20 points (unless score is 0) for living with active indoor smoker in home |
|  | **Sleep health** | **Measuremen**t: Self-reported average hours of sleep per night  **Example tools for measurement**:  “On average, how many hours of sleep do you get per night?” Consider objective  sleep/actigraphy data from wearable technology, if available | **Metric**: Average hours of sleep per night  **Scoring**:  Points Level  100 7 – <9  90 9 - <10  70 6 - <7  40 5 - <6 or ≥10  20 4 - <5  0 <4 |
| **Health Factors** | **Body mass index** | **Measurement**: Body weight (kg) divided by height squared (m^2^)  **Example tools for measurement**:  Objective measurement of height and weight | **Metric**: Body mass index (kg/m^2^)  **Scoring**:  Points Level  100 <25  70 25.0 – 29.9  30 30.0 – 34.9  15 35.0 – 39.9  0 ≥40.0 |
|  | **Blood lipids** | **Measurement**: Plasma total and HDL-cholesterol with calculation of non-HDL-cholesterol  **Example tools for measurement**:  Fasting or non-fasting blood sample | **Metric**: Non-HDL-cholesterol (mg/dL)  **Scoring**:  Points Level  100 <130  60 130 – 159  40 160 – 189  20 190 – 219  0 ≥220  If drug-treated level, subtract 20 points |
|  | **Blood glucose** | **Measurement**: Fasting blood glucose or casual hemoglobin A1c  **Example tools for measurement**:  Fasting (FBG, HbA1c) or nonfasting (HbA1c) blood sample | **Metric**: Fasting blood glucose (mg/dL) or  Hemoglobin A1c (%)  **Scoring**:  Points Level  100 No history of diabetes and FBG <100 (or HbA1c < 5.7)  60 No diabetes and FBG 100 – 125 (or HbA1c 5.7-6.4) (Pre-diabetes)  40 Diabetes with HbA1c <7.0  30 Diabetes with HbA1c 7.0 – 7.9  20 Diabetes with HbA1c 8.0 – 8.9  10 Diabetes with Hb A1c 9.0 – 9.9  0 Diabetes with HbA1c ≥10.0 |
|  | **Blood pressure** | **Measurement**: Appropriately measured systolic and diastolic blood pressure  **Example tools for measurement**:  Appropriately sized blood pressure cuff | **Metric**: Systolic and diastolic blood pressure (mmHg)  **Scoring**:  Points Level  100 <120/<80 (Optimal)  75 120-129/<80 (Elevated)  50 130-139 or 80-89 (Stage I HTN)  25 140-159 or 90-99  0 ≥160 or ≥100  Subtract 20 points if treated level |
